# FOXN1 deficient nude severe combined immunodeficiency

**DOI:** 10.1186/s13023-016-0557-1

**Published:** 2017-01-11

**Authors:** Ioanna A. Rota, Fatima Dhalla

**Affiliations:** 1 Developmental Immunology Group, Department of Paediatrics, University of Oxford, Oxford, UK; 2 Department of Clinical Immunology, Oxford University Hospitals, Oxford, UK

**Keywords:** Immunodeficiency, Thymus, T-cell, Alopecia, Nail dystrophy, FOXN1

## Abstract

Nude severe combined immunodeficiency is a rare inherited disease caused by autosomal recessive loss-of-function mutations in *FOXN1*. This gene encodes a transcription factor essential for the development of the thymus, the primary lymphoid organ that supports T-cell development and selection. To date nine cases have been reported presenting with the clinical triad of absent thymus resulting in severe T-cell immunodeficiency, congenital alopecia universalis and nail dystrophy. Diagnosis relies on testing for *FOXN1* mutations, which allows genetic counselling and guides therapeutic management. Options for treating the underlying immune deficiency include HLA-matched genoidentical haematopoietic cell transplantation containing mature donor T-cells or thymus tissue transplantation. Experience from other severe combined immune deficiency syndromes suggests that early diagnosis, supportive care and definitive management result in better patient outcomes. Without these the prognosis is poor due to early-onset life threatening infections.

## Background

Nude severe combined immunodeficiency (SCID) is a rare inherited syndrome caused by a functional deficiency of FOXN1, a transcription factor essential for the development and function of thymic epithelial cells (TECs) [1–3].

The thymus is the primary lymphoid organ responsible for the development of T lymphocytes from bone marrow derived haematopoietic precursors [4]. The unique three-dimensional structure of TECs forms the appropriate physiological microenvironment for the generation T-cells able to effect immune responses against foreign pathogens whilst being tolerant to the body’s own proteins (designated “self”) [5]. The study of loss-of-function mutations in *Foxn1* in animal models has shown its critical importance in TEC differentiation, homeostatic maintenance and T-cell lymphopoiesis [3, 6–9].

Absent thymus (athymia), alopecia universalis (AU) and nail dystrophy were first noted in 1966 in a spontaneously occurring phenotype in the so-called nude mouse [9–11]. The molecular cause was identified in 1994 to be due to an autosomal recessive deletional mutation in the *whn* gene*,* later renamed *Foxn1* [7, 12]. Thirty years after its first description in mice, the human counterpart of the nude phenotype was reported in two sisters presenting with early-onset severe immunodeficiency associated with congenital alopecia and nail dystrophy [1, 2].

FOXN1 is required for the development of epithelial cells in the thymus, the skin, hair and nails [7, 13–19]. As the developmental defect of TECs results in a lack of regular T-cell development and selection, FOXN1 deficiency has been classified as a rare form of severe combined immunodeficiency (SCID) with absent or low T-cells (i.e. a T^-/low^B^+^NK^+^ SCID). SCID syndromes are an aetiologically heterogeneous group of genetic disorders, defined by defects in T-cell development and function and a variable impact on the development of B- and NK-cells [20]. Consequently patients are unable to produce protective immune responses and present in early infancy with life-threatening infections [20]. Nude SCID is an example of a SCID syndrome that is not due to mutation of a gene expressed in hematopoietic cells but rather constitutes an abnormality of the thymic stromal cell compartment, namely TECs, essential for normal T-cell development [21]. As with other SCIDs, early diagnosis and management is critical in order to prevent accumulation of end-organ damage due to severe infections [22].

## Review

### Disease name/synonyms

Nude SCID [2, 23] is also known as FOXN1 deficiency [23], alymphoid cystic thymic dysgenesis (ORPHA169095) [24], severe T-cell immunodeficiency, congenital alopecia, nail dystrophy syndrome (MIM601705) [1] and Winged helix deficiency [2].

### Epidemiology

Nude SCID is very rare with an estimated incidence of <1/1,000,000. Only nine cases have been reported in the literature to date. Six patients originated from Acerno in southern Italy; all had the same homozygous founder mutation (R255X) carried by 6.52% of the village’s inhabitants [25]. An identical mutation was later identified in a Portuguese child born to consanguineous parents [23]. Two additional mutations have been identified in single patients of mixed French/African (R320W) and consanguineous Lebanese origin (S188fs) [23, 26].

### Clinical description

The human nude SCID phenotype is characterised by the clinical triad of athymia and resultant SCID, congenital AU and nail dystrophy (Table 1) [1, 23, 25–27].

**Table 1:** Table of reported cases of FOXN1 deficiency

Patient (Sex)	Mutation	Ethnic origin	Clinical Phenotype						Immunological Phenotype				Treatment	Outcome	Ref.
			Congenital AU	Nail dystrophy	Recurrent Infections	OS	FTT	Other	Total lymph-ocytes	T	B	NK			
1 (F) Sib. of patient 2	R255X	Acerno, Southern Italy	+	+	+	+ (2 m)	+ (2 m)	- Bilateral epicanthic folds	↑	↓ - Greater ↓ in CD4 vs. CD8 - Abnormal proliferation - Absent thymus on CXR	↔ - Defective Ab production	↔	Supportive	Died aged 12 m (resistant bronchopneumonia)	[1, 2]
2 (F) Sib. of patient 1	R255X	Acerno, Southern Italy	+	+	+ - Pyogenic - Respiratory tract	Mild (2 m)	+	- Bilateral epicanthic folds	↔	↓ - Greater ↓ in CD4 vs. CD8 - Abnormal proliferation - Absent thymus on CXR	↔	↔	HLA matched Sib. BMT 5 m	6y post BMT: - Failed to reconstitute naïve CD4 T-cells - Abnormal T-cell proliferation & TCR repertoire - But infection free - Persistent AU and nail dystrophy	[1, 2, 30]
3 to 6	R255X	Acerno, Southern Italy	+		+									Died early childhood (severe infections)	[25]
7 (F)	R255X	Portugal	+	+	+ - Atypical mycobacteria (BCG, *M. bovis*) - Rotavirus	Mild (3 m)	+		↔	↓ - Greater ↓ in CD4 vs. CD8 - ≤1% naïve CD4 T-cells - Oligoclonal TCR repertoire - ↓TRECs - Abnormal proliferation	↔ - Defective Ab production	↔	- HLA mismatched thymus transplant (14 m) - Ig infusions (pre-transplant)	5y post thymus transplant: - T-cell compartment successfully reconstituted - Infection free - Normal Ab production	[23, 63]
8 (M)	R320W	France/ Africa	+	+	+ - Respiratory tract - HHV6				↓	↓ - Absent T-cells & proliferation	↓	↔	- HLA mismatched thymus transplant (9 m) - Ig infusions (pre-transplant)	3y post thymus transplant: - T-cell compartment successfully reconstituted - Infection free - Normal Ab production - Thyroid autoimmunity & vitiligo - Persistent AU and nail dystrophy	[23, 27]
9(F)	S188fs	Lebanon	+			+ (1 m)				↓ - ≤1% naïve CD4 T-cells - Absent recent thymic emigrants - Abnormal proliferation	↔	↔	HLA matched sib. HSCT (5 m)	Died (complications post HSCT)	[26]

Key: *AU* alopecia universalis, *OS* Omenn Syndrome, *FTT* failure to thrive, *T* T-cells, *B* B-cells, *NK* NK-cells, *Ref*. References, *F* female, *M* male, *Sib* sibling, + present, − absent, *m* months old, *y* years, ↑ increased count, ↓ decreased count, ↔ normal count, *CD* cluster of differentiation, *CXR* chest x-ray, *Ab* antibody, *HLA* Human leucocyte antigen, *BMT* bone marrow transplant, *TCR* T-cell receptor, *BCG* Bacillus Calmette–Guérin, *HHV6* Human herpes virus 6, *TRECs* T-cell receptor excision circles, *Ig* Immunoglobulin, *HSCT* Haematopoietic stem cell transplant

All reported patients presented in the first months of life with severe, recurrent, life-threatening infections [1, 23, 25] reflecting their severely impaired T-cell-mediated immune response to viral, fungal and opportunistic infections as well as live vaccines [1, 23, 28, 29]. Although B-cells are typically present in normal numbers, antibody production is compromised in the absence of T-cell help [1, 23, 29] rendering patients susceptible to infections with encapsulated bacteria [1, 23, 29, 30]. Patients with nude SCID may have features of Omenn Syndrome (OS) [1, 23, 26], an inflammatory condition caused by expansions auto-reactive T-cells in the setting of SCID and characterised by erythroderma, hepatosplenomegaly, lymphadenopathy, diarrhoea and failure-to-thrive [31]. A detailed description of the immunological phenotype can be found in Tables 1 and 2 and in the section on diagnosis.

**Table 2:** Table of suggested diagnostic tests and investigations with expected findings

Category	Test(s)	Expected findings	Ref.
Genetic	- *FOXN1* sequencing - PCR for previously reported *FOXN1* mutations	- Homozygous *FOXN1* mutation - Previously reported mutations: R255X, R320W, S188fs	[2, 23, 25, 26]
Basic Immunology	Differential white cell count	- Total lymphocyte count ↓/↔/↑ - ↑ Eosinophils in Omenn syndrome	[1, 23]
	Lymphocyte subpopulations	- ↓ T-cell count (greater reduction in CD4+ T-cells Vs. CD8+) - ↔B-cell count (although ↓ in 1 reported case) - ↔/↑ NK-cell count	[1, 23, 26]
	Serum Immunoglobulins	↔/↓ - ↑ IgE in Omenn syndrome	[23]
Specialised Immunology	TRECs	Severely ↓ or absent	[23]
	Recent thymic emigrants (CD4 + CD31 + CD45RA+)	Severely ↓ or absent	[26]
	Markers of T-cell memory (CD45RA & CD45RO) and activation (HLA-DR)	- Severely ↓ naïve (CD45RA+) T-cells - ↑ memory (CD45RO+) T-cells - ↑ HLA-DR+ in Omenn syndrome	[23, 26, 30]
	T-cell proliferation to mitogens	- ↓ in response to anti-CD3 &/or PHA - May be normal in response to PMA and ionomycin	[1, 23, 26]
	T-cell receptor repertoire via flow cytometry or spectratyping	Oligoclonal	[23, 26]
	Specific antibodies to exposure and immunisation antigens	↓	[1]
Thoracic imaging	Chest x-ray/ultrasound scan/MRI	- Absent thymus - May show evidence of respiratory tract infection	[1]

Key: *Ref.* References, *PCR* polymerase chain reaction, ↑ increased, ↓ decreased, ↔ normal, *CD* cluster of differentiation, *Ig* immunoglobulin, *TRECs* T-cell receptor excision circles, *HLA* Human leucocyte antigen, *PHA* Phytohaemagglutinin, *PMA* phorbol myristate acetate, *MRI* magnetic resonance imaging

Dermatological features include congenital alopecia affecting the scalp, eyebrows and eyelashes, and nail dystrophy. The latter most frequently features proximal arciform leukonychia and koilonychia, although canaliform dystrophy and Beau’s lines have been noted [32]. Nail dystrophy has also been found in heterozygous carriers of *FOXN1* mutations [32].

CNS defects have only been described in two fetuses from a single kindred in the highly consanguineous village of Acerno. One displayed anencephaly and spina bifida [13], the other had milder abnormalities including an enlarged interhemispheric fissure and absence of the cavum septi pellucidi and corpus callosum [14].

### Aetiology

Following the fist description of nude SCID [1], linkage analysis and sequencing of the *FOXN1* gene in the two index cases, revealed a homozygous nonsense mutation leading to a premature stop codon at amino acid 255 (R255X) [2]. Two additional autosomal recessive *FOXN1* mutations (R320W and S188fs) have since been described [23, 26].

**Fig. 1:**
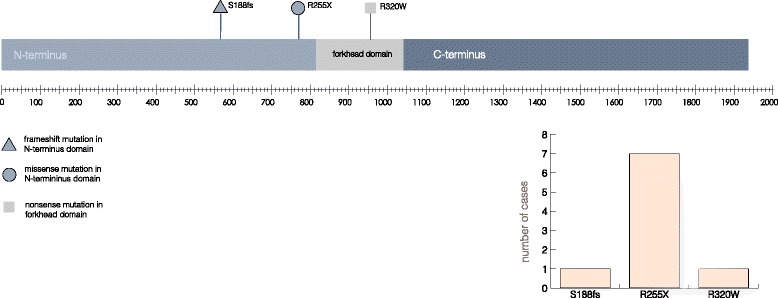
FOXN1 secondary/domain structure annotated with locations of mutations known to cause nude SCID in humans. The scale bar represents position in base pairs within the cDNA sequence. Also included is a bar chart showing number of cases described for each mutation

The forkhead box N1 (FOXN1) protein is a transcription factor expressed in epithelial cells of the thymus, skin, hair follicles and nail bed [13, 15, 33]. The precise molecular mechanisms of FOXN1 function are not completely understood. It is thought to be activated by phosphorylation, translocate to the nucleus [34–36], bind DNA through its forkhead domain (Fig. 1) [12, 37, 38], and promote the transcription of genes that control the development of epithelial cells [3]. Experimental models have demonstrated that the N-terminal aspect of FOXN1 is critical for murine TEC differentiation and the C-terminus is required for transcriptional activation of target genes [37, 39, 40].

The reported human *FOXN1* mutations are located in different domains of the molecule (Fig. 1), however all are thought to result in loss of function. The R255X and S188fs mutations, located in the N-terminus, both cause a premature stop codon predicted to result in non-sense mediated decay of the mRNA [2, 26]. The R320W mutation lies in the evolutionary conserved forkhead domain and is thought to impair the ability of the mutated protein to bind DNA and thus regulate the transcription of target genes [23].

T-cells are derived from blood-borne haematopoietic precursors that seed the thymus where they develop within a meshwork of stromal cells built primarily by TECs [41]. TECs secrete, in a FOXN1 dependent manner, several chemokines, CCL25, CCL21, CXCL12, that are required for attracting haematopoietic progenitors to the developing thymus [42]. These progenitors subsequently commit to a T-cell fate with the support of TEC-derived molecules such as the notch ligand DLL4, which is also transcriptionally regulated by FOXN1 [43]. Following an initial round of expansion, developing T-cells are subjected to selection processes, termed “positive” and “negative” selection, which are driven by recognition of MHC–self antigen complexes presented on the surface of TECs [5, 41]. This interaction leads to the selection of a T-cell repertoire that is self-tolerant but able to respond to foreign antigens [5, 41]. The developing T-cells then undergo a final maturation process before exiting to the peripheral circulation as single positive CD4 or CD8 naïve T-cells [5, 41]. FOXN1 is a core transcriptional regulator essential for TEC differentiation, maintenance and function [3, 44, 45]. It is now known to control the expression of hundreds of genes in TECs that support intrathymic T-cell development [3]. In addition to *CCL25* [42], *CXCL12* [3], and *DLL4* [43], FOXN1 positively regulates the expression of a number of genes involved in antigen processing and presentation [3]. Lack of functional FOXN1 in TECs therefore disrupts normal thymic organogenesis and the ability to support T-cell lineage commitment, development and selection [8, 10, 11, 15].

In the skin and its appendages FOXN1 is expressed in epithelial cells that have stopped proliferating and are in the process of terminal differentiation [15, 33]. Studies in mouse keratinocytes suggest that FOXN1 controls the expression of protein kinase B and C, molecules that are involved in cell survival, metabolism and cell cycle progression [46, 47]. As a consequence, loss-of-function mutations disrupt the balance between normal growth and differentiation of these cells [15, 17–19]. Humans and mice with FOXN1 deficiency have numerically normal hair follicles that give rise to hairs with an abnormal shaft causing them to curl and break off at the level of the skin surface leading to alopecia [9, 48].

The role of FOXN1 in CNS development is not confirmed. Neurodevelopmental defects have not been reported in mouse models and, given that the only two fetuses with neurological abnormalities came from the same family within a closed population [13, 14], it is possible that another genetic aetiology was responsible for their neurological features, however this has not been formally investigated.

### Diagnosis

Nude SCID due to FOXN1 deficiency should be suspected in infants presenting with clinical and/or laboratory evidence of immunodeficiency associated with congenital AU and nail dystrophy [1, 2, 23, 25, 26, 49].

Population-based newborn screening (NBS) programmes for SCID have been introduced in several countries [50–52]. Polymerase chain reaction (PCR) on DNA extracted from Guthrie card blood spots is used to quantify circularised DNA by-products generated during TCR formation in the thymus, called T-cell receptor excision circles (TRECs) [53, 54]. Deficient levels of TRECs identified during NBS indicate T-cell deficiency requiring further investigation. It is predictable that FOXN1 deficiency will be detectable on the basis of absent/low TRECs although, as yet, there are no reports in the literature of patients identified via NBS. Indeed, Infants with FOXN1 deficiency have been shown to have very low TREC levels [23] and lack other markers of thymic T-cell output [26]. In addition, NBS has been able to identify patients with severe T-cell deficiency due to other primary thymic defects including DiGeorge (DGS) and CHARGE syndrome [52, 55].

Infants with suspected nude SCID should be immediately referred to a specialist centre experienced in and equipped for specialised immunological tests and management of severe immunodeficiencies [28]. This is important as early diagnosis and treatment have been shown to greatly impact upon outcomes, including survival, in children with SCIDs [56]. Diagnostic tests and further investigations are detailed in the text below and summarised along with expected findings in Table 2.

Definitive diagnosis relies on testing for *FOXN1* mutations [2] and is essential in order to guide patient management and genetic counselling [23, 26]. If the clinical suspicion is high this may be achieved by a targeted approach using single gene Sanger sequencing or screening for described mutations [2, 23, 25, 26]. Alternatively, next generation sequencing techniques may be used including targeted sequencing panels, which are increasingly available for the molecular diagnosis of patients presenting with primary immune deficiency [57–61].

Basic immunological assessment should include enumeration of total lymphocytes, lymphocyte subpopulations (T-, B-, and NK-cells), and serum immunoglobulins [22, 62]. Results should be interpreted alongside age-specific reference ranges. Total lymphocyte count may be normal, decreased or increased [1, 23]. However, patients have universally shown low T-cell counts [1, 23, 26], with CD4+ T-cells more severely affected than CD8 + [1, 23]. NK- and B-cells are expected to be present, although the latter are poorly functional in terms of specific antibody production [1, 23, 26].

More specialised investigations include analysis of T-cell subpopulations and receptor repertoire, markers of thymic T-cell output, and T- and B-cell function [22, 62]. Patients with FOXN1 deficiency have been shown to lack evidence of efficient thymic T-cell output with increased double negative (CD4-CD8-) T-cells in the peripheral blood [23, 63], and severe reductions in TRECs [23], CD31+ recent thymic emigrants [26], and CD45RA+ naïve CD4+ T-cells resulting in skewing towards a CD45RO+ memory phenotype [23, 26, 30]. Their T-cells show reduced in vitro proliferation and an oligoclonal TCR repertoire [1, 23, 26]. Those presenting with OS may have eosinophilia, elevated serum IgE and presence of activated (HLADR+), oligoclonal T-cells [22, 62].

Thoracic imaging should be performed to document thymic hypo-/aplasia [1, 22]. Patients should be actively screened for viral, fungal and bacterial infections via microbiological examination of respiratory secretions and stools, and imaging; blood should also be tested for the presence of Epstein Barr (EBV) and cytomegalovirus (CMV) nucleic acid [64]. It is important to note that serological tests are unreliable due to poor B-cell function.

### Differential diagnosis

Although the triad of congenital AU, nail dystrophy and athymia is highly indicative of FOXN1 deficient nude SCID, there are several differential diagnoses that warrant consideration (Table 3). These include alternative causes of SCID, combined immune deficiency (CID) and OS that have a similar immunophenotype (i.e. T^-/low^B^+^NK^+^), other primary thymic defects [49], and dyskeratosis congenita (DC). However, in DC differentiating clinical features such as abnormal skin pigmentation and oral leucoplakia are often present [48, 49, 65].

**Table 3:** Table of differential diagnoses

Disease	Genetic defect(s)	Orphanet Number	Typical clinical phenotype	Thymus	Immunophenotype			Refs.
					T	B	NK	
Nude SCID	*FOXN1* (AR)	ORPHA169095	Congenital AU, nail dystrophy, OS, early onset severe recurrent infections	Absent	↓	↔	↔	[2, 23, 26]
T-B + NK+ SCIDs	*IL-7Rα* (AR)	ORPHA169154	FTT, diarrhoea, rash, early onset severe recurrent infections. Chronic EBV & EBV-driven lymphoma (coronin1a deficiency)	Present/Absent	↓	↔	↔ (may be ↓ in coronin1a & CD45 deficiency)	[49, 80–89]
	*CORO1A* (AR)	ORPHA228003						
	*CD45* (AR)	ORPHA169157						
	*CD3δ* (AR)	ORPHA169160						
	*CD3ε* (AR)	ORPHA169160						
	*CD3ζ* (AR)	ORPHA169160						
CIDs	MHC II deficiency: *CIITA, RFX5, RFXAP, RFXANK* (AR)	ORPHA572	FTT, chronic diarrhoea, autoimmunity, recurrent severe infections of respiratory and gastrointestinal tracts	ND	↓ CD4+ T-cells	↔	↔	[49, 90–93]
	*MAGT1* (XL)	ORPHA317476	Splenomegaly, chronic EBV viraemia, EBV-driven lymphoma, recurrent infections	ND	↓ CD4+ T-cells	↔	↔	[49, 94]
	*LCK* (AR)	ORPHA 280142	FTT, diarrhoea, autoimmunity, recurrent severe infections	ND	↓ CD4+ T-cells	↔	↔	[49, 95]
Omenn Syndrome	E.g. *IL-7Rα, RMRP* (AR) *CHD7* (AD) Atypical complete DGS	ORPHA39041	Erythroderma, eczema, diarrhoea, hepatosplenomegaly, lymphadenopathy, eosinophilia, high serum IgE, early onset severe recurrent infections	Normal/Hypoplastic/Absent	↔/↓	↔	↔	[49, 81, 96–100]
DiGeorge Syndrome	22q11.2 deletion 90% of cases (de novo/AD) 10p deletions	ORPHA567	Heart defects, hypoparathyroidism, facial dysmorphism, developmental delay. Variable infection susceptibility: No/mild infections (partial DGS) to severe recurrent infections (complete DGS)	Normal/Hypoplastic (partial DGS)/Absent (complete DGS)	↔/↓	↔	↔	[49, 69, 101]
CHARGE Syndrome	*CHD7 (70% of cases)* (de novo or AD)	ORPHA138	Coloboma, heart defects, choanal atresia, retardation of growth/development, ear abnormalities/deafness. Variable infection susceptibility	Normal/Hypoplastic/Absent	↔/↓	↔	↔	[49, 97, 102, 103]
Dyskeratosis Congenita	*TERC, TERT, TINF2, RTEL1* (AD) *TERT, CTC1, RTEL1, WRAP53, NHP2, NOP10* (AR) *DKC1I* (XL)	ORPHA1775	Alopecia, nail dystrophy, reticular hyper- and hypo-pigmentation, oral leukoplakia, recurrent sinopulmonary or opportunistic infections	ND	↔/↓	↔/↓	↔/↓	[49, 104–107]

Key: *Ref.* References, *SCID* severe combined immunodeficiency, *AR* autosomal recessive, *AD* autosomal dominant, *XL* x-linked, *AU* alopecia universalis, *OS* Omenn syndrome, ↑ increased, ↓ decreased, ↔ normal, *FTT* failure-to-thrive, *EBV* Epstein-Barr virus, *CID* combined immunodeficiency, *CD* cluster of differentiation, *Ig* immunoglobulin, *DGS* DiGeorge syndrome

### Genetic counselling and antenatal diagnosis

Once a molecular diagnosis is ascertained, parental carrier status should be assessed. In highly consanguineous populations, testing for carrier status could also be extended to the wider family [25]. As an autosomal recessive disease, the risk of disease transmission in future pregnancies is 1 in 4 if both parents are carriers. Antenatal diagnosis can be achieved via chorionic villus sampling or amniocentesis [25, 66]. Where parents decide to continue with an affected pregnancy, this will allow preparation for immediate supportive and early definitive management of the underlying immune deficiency in a specialist centre [56].

### Management

Infants with suspected nude SCID require prompt referral to a specialist centre experienced in management of SCID. Management of such cases involves supportive care, which aims to optimise the patient’s clinical condition before timely institution of definitive treatment to correct the underlying immune deficiency [64].

Prophylaxis and early treatment of infections is of upmost importance and has been shown to improve outcomes in other forms of SCIDs [28, 67]. This involves isolation in a laminar flow room, prophylaxis against *Pneumocystis jiroveci* pneumonia, fungal and viral infections, and immunoglobulin replacement [28, 62, 64, 67]. Live vaccines are contraindicated and anti-mycobacterial treatment should be initiated in those immunised with BCG before an immunodeficiency was suspected [28, 62, 64]. If blood products are required these should be CMV negative, irradiated and depleted of leucocytes [28, 62, 64, 68]. In the setting of OS careful immunosuppression may be required [64].

Of the four patients that have received treatments aimed at correcting the underlying immune deficiency, two received HLA-matched sibling/genoidentical haematopoietic cell transplants (HCT) at the age of 5 months [1, 26, 30], and two had thymic transplants at 9 and 14 months of age [23].

One of the HCT recipients died following post-transplant complications [26], whereas the other was alive and infection-free when assessed 6 years later likely due to the presence of mature donor T-cells with proliferative capacity present in the bone marrow graft [30, 69]. Experience from complete DGS suggests that HCT is unlikely to result in high quality immune reconstitution in the context of an underlying thymic stromal cell defect [30, 69]. However, patients treated with HLA-matched sibling HCT have better outcomes compared to those treated with matched unrelated transplants [70]. In a multicentre retrospective study on the outcomes of 17 patients with complete DGS treated with HCT overall survival was 41% after 4–11.5 years of follow-up. However, in the subgroup that received transplants from HLA-matched sibling donors overall survival was significantly better at over 60% [70]. There are several possible reasons for this: for example, the need for serotherapy using antibodies directed against T-cells in patients treated with matched unrelated transplants is likely to preclude the survival of mature donor T-cells present in the graft necessary to provide cellular immunity in the absence of a functional thymus. In addition, it has been noted that graft versus host disease is particularly severe in patients with athymia [70].

Given that FOXN1 is expressed in TECs and not haematopoietic cells, establishing a functional thymic stromal environment is expected to provide more complete and long-lasting immune reconstitution [23, 26]. This can be achieved via transplantation into the quadriceps muscle of non-HLA matched thymic tissue obtained from infants undergoing corrective cardiac surgery [71]. This highly specialist treatment is currently limited to two centres worldwide [69]. Reconstitution of successful T-cell lymphopoiesis was achieved in both FOXN1 deficient cases treated with thymic transplantation as evidenced by T-cell count, and the presence of TREC positive naïve CD4+ T-cells, and CD31+ recent thymic emigrants in the peripheral blood. The newly generated T-cells proliferate normally, display a diverse TCR repertoire, and are able to support the production of specific antibodies directed against T-cell dependent antigens [23, 63]. Both patients cleared infections present pre-transplantation and remained infection-free 3–5 years later. However, one patient developed autoimmune hypothyroidism and vitiligo [23, 27]. Precedence for the use of thymic transplantation in patients with FOXN1 deficiency comes from experience in complete DGS, where patients also have an intrinsic thymic stromal defect that precludes normal T-cell development [69, 70, 72]. Outcomes after thymic transplantation for complete DGS are at least as good as HCT with respect to overall survival (over 70%), and the quality of immune reconstitution is superior [69–73]. T-cell reconstitution after thymic transplantation however takes several months and autoimmune diseases are observed in a third of patients principally affecting the thyroid [69, 71, 73].

In summary, from the available evidence, the following recommendations can be made in order to aid in the selection of the most appropriate definitive treatment for individual patients with nude SCID. HCT containing mature donor T-cells should only be offered to patients with an HLA-matched genoidentical sibling donor; this treatment approach may be particularly important in situations where thymic transplantation is not readily available or in the context of pre-existing systemic viral infection, where rapid recovery of T-cell mediated immunity is required [69, 70, 74]. Alternatively, thymic transplantation could be used, without the need for HLA-matching, provided that it is accessible in a timely manner and that rapid T-cell recovery is not required [74, 75]. If an HLA-matched sibling donor is not available for HCT, however, evidence from DGS suggests that outcomes are likely to be superior with thymic transplantation [70].

Important developments within the field of regenerative medicine may provide strategies for the definitive management of thymic stromal cell defects in the future. Induced pluripotent stem cells (iPSCs) have been used to successfully generate thymic organoids capable of supporting in vivo T-cell development in mouse models, including nude mice [76–79]. Although HLA-matching is not essential for transplantation of thymic tissue [75], this technology could be combined with gene therapy in order to allow the transplantation of autologous thymic organoids generated from gene corrected iPSCs.

### Prognosis

Early diagnosis, supportive care and definitive treatment results in improved outcomes for patients with SCID [56]. All reported nude SCID patients in whom definitive treatments could not be established succumbed to infections very early in childhood [1, 25].

### Unresolved questions

It remains unclear whether any relevant genotype-phenotype correlation exists that could explain the variation in immunological findings observed. The patient with a missense mutation in the forkhead domain (R320W) demonstrated complete absence of circulating T-cells [23], whereas patients with mutations in the N-terminus that lead to premature stop codons (R255X and S188fs) [2, 23, 26], have a less severe immunological phenotype and retain a limited number of peripheral T-cells. A possible explanation for the milder phenotype in the latter could be re-initiation of transcription from an alternative start codon downstream of the mutations. Indeed two such possible alternative start codons exist and, if formed, the resulting transcripts would have intact DNA binding and transcriptional activation domains and therefore could translate into partially functional N-terminally truncated proteins. In contrast, the R320W mutation is thought to impair binding of the mutated FOXN1 protein to DNA and thus abrogate its ability to regulate the transcription of target genes [23]. However, with such few cases reported and in the absence of experimental evidence to confirm or refute the above, it is difficult to draw firm conclusions concerning possible genotype-phenotype correlations and their mechanisms.

## Conclusions

Nude SCID caused by FOXN1 deficiency should be suspected in infants presenting with severe T-cell immunodeficiency associated with congenital AU and nail dystrophy. Prompt diagnosis, supportive care and referral to a specialist centre for definitive treatment are of paramount importance in order to ensure best possible outcomes [56].

## Abbreviations

Ab: Antibody
AU: Alopecia Universalis
BCG: Bacillus Calmette–Guérin
BMT: Bone marrow transplant
CD: Cluster of differentiation
CHARGE: Coloboma, heart defects, atresia of the choanae, retardation of growth/development, ear abnormalities/deafness
CID: Combined immunodeficiency
CNS: Central nervous system
CXR: Chest x-ray
DGS: DiGeorge syndrome
DLL4: Delta-like ligand 4
FOXN1: Forkhead box N1
FTT: Failure-to-thrive
HCT: Haematopoietic cell transplant
HLA: Human leucocyte antigen
HSCT: Haematopoietic stem cell transplant
Ig: Immunoglobulin
MRI: Magnetic resonance imaging
NBS: Newborn screening
NK: Natural killer
OS: Omenn syndrome
PCR: Polymerase chain reaction
PHA: Phytohaemagglutinin
PKC: protein kinase C
PMA: Phorbol myristate acetate
SCID: Severe Combined Immunodeficiency
Sib: Sibling
TCR: T-cell receptor
TECs: Thymic epithelial cells
TRECs: T-cell receptor excision circles
